# Association of *CYP1A1* and *GSTM1* Polymorphisms With Oral Cancer Susceptibility: A Meta-Analysis

**DOI:** 10.1097/MD.0000000000000895

**Published:** 2015-07-13

**Authors:** Haitao Liu, Jinlin Jia, Xuemei Mao, Zhiyong Lin

**Affiliations:** From the Department of Stomatology (HL, JJ, XM), People's Hospital of Dongying, Dongying; and Department of Stomatology (ZL), Shandong Provincial Hospital Affiliated to Shandong University, Shandong, China.

## Abstract

Our meta-analysis was aimed to evaluate the association of *CYP1A1* and glutathione-*S*-transferase M1 (*GSTM1*) polymorphisms with oral cancer susceptibility.

The related articles were searched in PubMed, Embase, and CNKI databases. Fifty eligible studies were included in our meta-analysis. Odds ratios (ORs) with 95% confidence intervals (CIs) were used to evaluate the relationship of *CYP1A1* (rs4646903 and rs1048943) and *GSTM1* polymorphisms with oral cancer risk. A random-effects model or fixed-effects model was employed depending on the heterogeneity.

In overall analysis, *CYP1A1* rs4646903 polymorphism was associated with the risk of oral cancer (CC vs TT: OR 1.65, 95% CI 1.33–2.05; CC vs TC+TT: OR 1.77, 95% CI 1.48–2.11; C vs T: OR 1.17, 95% CI 1.07–1.28), whereas rs1048943 showed no obvious association with oral cancer susceptibility. Moreover, subgroup analysis by ethnicity demonstrated that rs4646903 and rs1048943 both related with increased risk of oral cancer in Asians. Moreover, the analysis based on source of control suggested that rs4646903 could increase the risk for oral cancer in both population- and hospital-based populations, whereas no remarkable relationship of rs1048943 with oral cancer susceptibility was observed. For *GSTM1* gene, null genotype appeared to be a risk factor for oral cancer (null vs present: OR 1.23, 95% CI 1.12–1.34), which was also proved in the subgroup analysis.

The results demonstrated that *CYP1A1* rs4646903 and null genotype of GSTM1 polymorphisms might serve as risk factors for oral cancer.

## INTRODUCTION

Oral cancer is one of the most common cancers in the world,^1^ the incidence of which has increased obviously in the last few years among different populations.^2,3^ It is generally considered that genetic polymorphisms and environmental factors including cigarette smoking, alcohol consumption, and betel quid chewing are of particular importance in the etiology of oral cancer.^4,5^

Genetic polymorphisms is prevalent and play a viral role in human diseases. Recently, the relationship of genetic polymorphisms and the risk of cancers have been researched widely. Among the genes, cytochrome *P450 1A1* (also known as *CYP1A1*) gene, located on chromosome 15, encodes aryl hydrocarbon hydrolase, which involves in metabolism of polycyclic aromatic hydrocarbons (PAHs).^6^ For *CYP1A1*, rs4646903 polymorphism, a T to C transition in the 3′ noncoding region (a thymine/cytosine point mutation), has been confirmed to be related with the high risk of lung and head and neck cancers.^7,8^ In addition, *CYP1A1* rs1048943 polymorphism, an amino acid substitution from isoleucine to valine at codon 462, shows the effects of enhancing catalytic activity and increasing the risk for lung cancer.^9,10^ For glutathione-*S*-transferase M1 (*GSTM1*), the polymorphism includes present genotype and null genotype, which are associated with abnormal function of GSTμ enzyme that is an important member in the detoxification of carcinogens in tobacco smoking.^11,12^ Moreover, the null genotype was reported to associate with increased risk of gastric, bladder, colon, and lung cancers.^13–16^ It is worth mentioning that CYP1A1, phase I enzyme, and GSTM1, phase II enzyme, could affect individual variability in the metabolism of chemical substances and finally affect the susceptibility to cancers through increasing the activity of xenobiotic metabolizing enzymes.^17–20^

Up to now, several epidemiological studies have focused on the association of *CYP1A1* and *GSTM1* polymorphisms with oral cancer susceptibility.^2,21–69^ However, the results remained conflicting. Therefore, the meta-analysis was carried out to gain more comprehensive evidences for the association.

## METHODS

### Search Strategy

The relevant articles were searched in PubMed, Embase, and CNKI databases using the keywords “*CYP1A1*” or “cytochrome P450 1A1,” “*GSTM1*” or “glutathione-*S*-transferase M1,” “polymorphism,” and “oral cancer.” The reference lists in retrieved papers were also screened manually for potential articles. All the selected studies should comply with the following inclusion criteria: case–control studies, studies about the association of *CYP1A1* and *GSTM1* polymorphisms with oral cancer susceptibility, and adequate data for estimating an odds ratio (OR) with 95% confidence interval (CI). When the same data existed in >1 publication, the largest or most recent publication was included. This study is a meta-analysis and does not involve populations; ethical approval was not required.

### Data Extraction

The following data were extracted from each study by 2 independent investigators: name of first author, publication date, country of origin, ethnicity, source of controls, genotyping methods, total number of cases and controls, genotype frequencies in case and control groups and Hardy–Weinberg equilibrium (HWE). Disagreements were solved by a discussion between the 2 investigators. The characteristics of the included articles were shown in Tables 1 and 2.

**TABLE 1:**
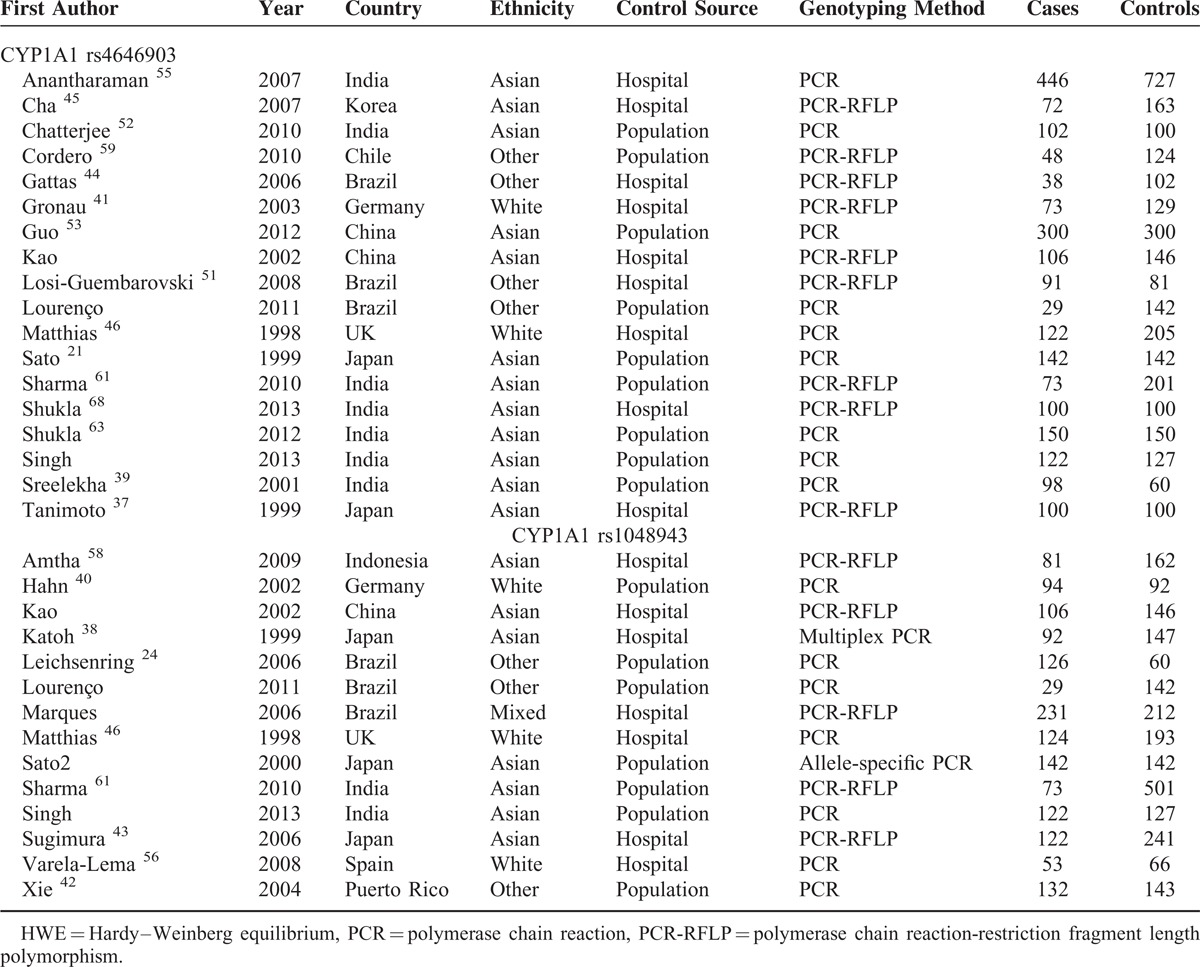
Characteristics of Studies on *CYP1A1* Polymorphisms

**TABLE 2:**
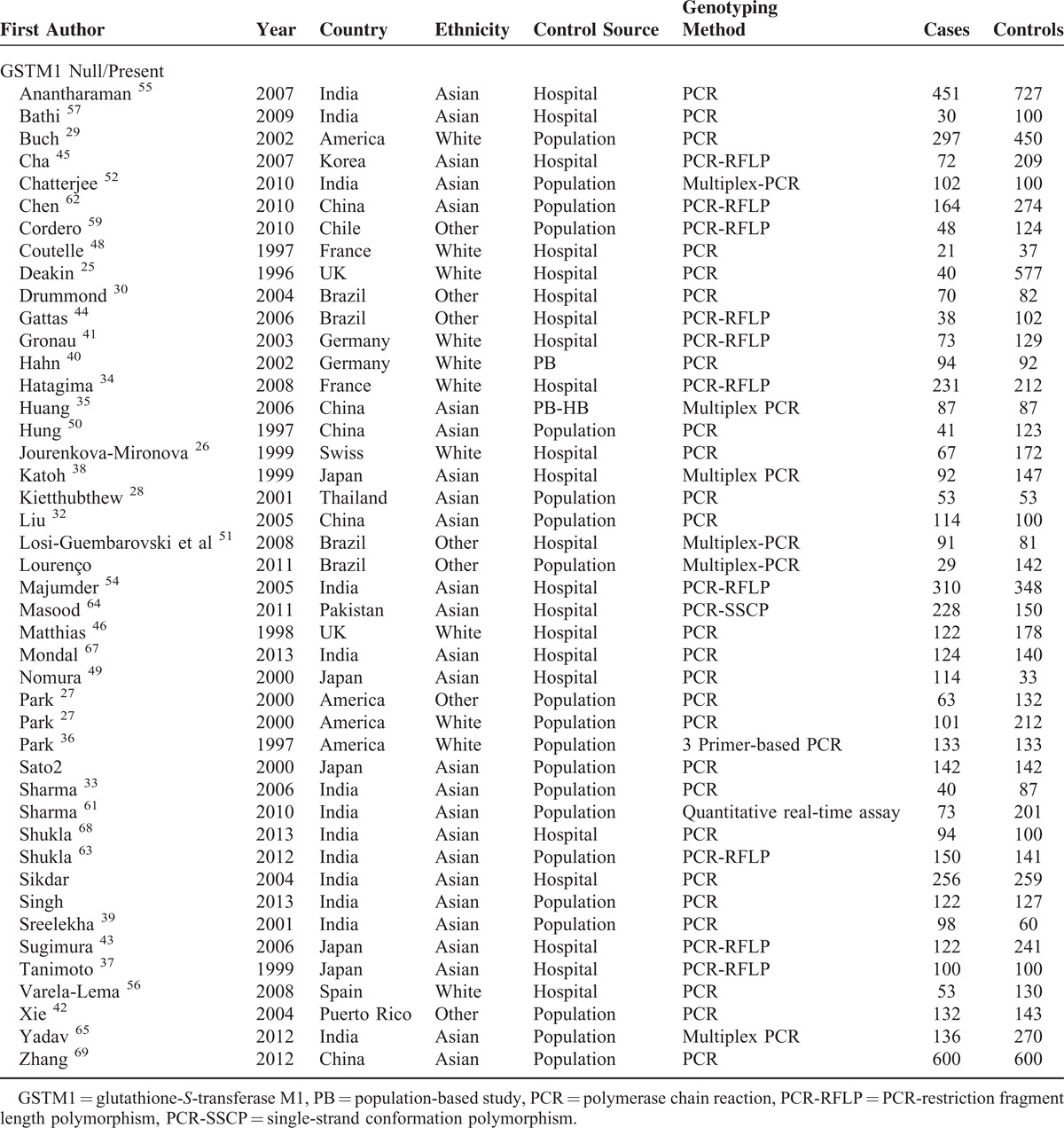
Principle Characteristics of Studies on *GSTM1* Null/Present

### Statistical Analysis

We applied crude ORs with corresponding 95% CIs to evaluate the association of *CYP1A1* and *GSTM1* polymorphisms with oral cancer susceptibility. Heterogeneity assumption was estimated by the χ^2^-based *Q* test. When *P* < 0.05, which indicated significant heterogeneity among studies, the pooled OR was calculated using the random-effects model; otherwise, the fixed-effects model was used. The pooled results of *CYP1A1* were analyzed under the following genetic models: 22 versus 11, 22 + 12 versus 11, 22 versus 11 + 12, 2 versus 1, and 12 versus 11. For *GSTM1*, null versus present and present versus null models were used. Sensitivity analysis was conducted to measure the stability of pooled results. Publication bias was assessed by Begg funnel plot and Egger test. HWE was checked by χ^2^ test. Statistical data were performed using the STATA software (version 12.0; Stata Corporation, Texas, Tex, USA).

## RESULTS

### Study Characteristics

**FIGURE 1:**
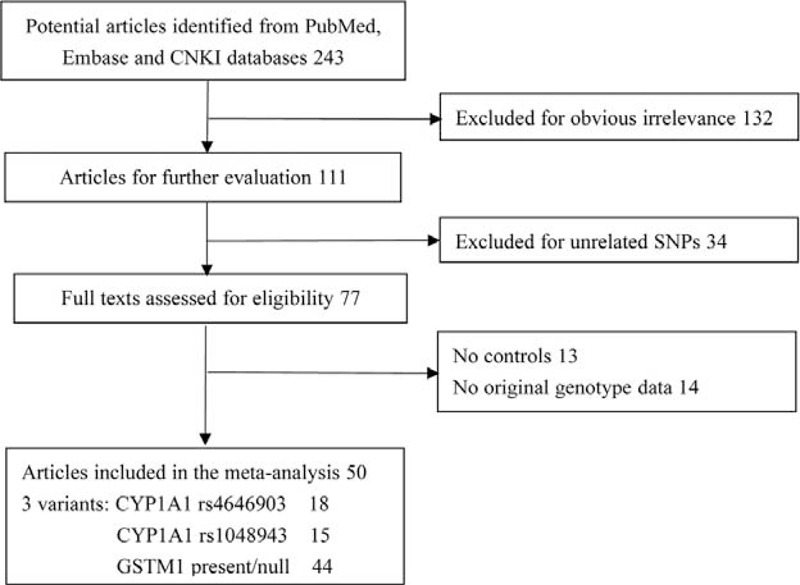
Flow diagram of included studies for the meta-analysis. CNKI = China National Knowledge Infrastructure, SNP = single nucleotide polymorphism.

As displayed in Figure 1, a total of 243 articles were searched through databases in which 132 articles were excluded for obvious irrelevance, 34 articles were excluded for unrelated single nucleotide polymorphisms (SNPs), and 27 articles were eliminated for not having controls and original genotype data. Finally, 50 articles were included in our meta-analysis.^2,21–69^

### Meta-Analysis

**FIGURE 2:**
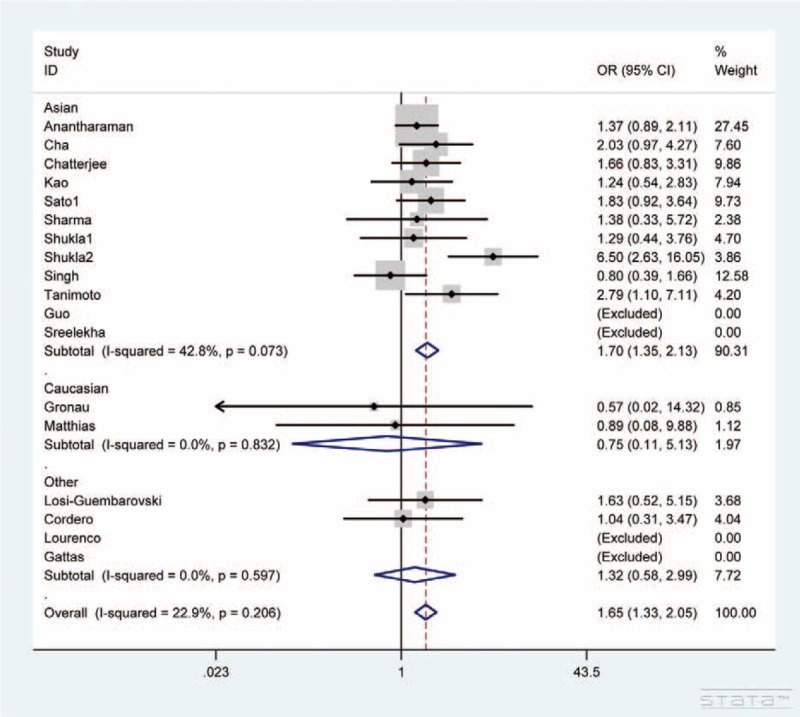
Forest plot of oral cancer susceptibility associated with CYP1A1 rs4646903 polymorphism under CC versus TT genetic model. CI = confidence interval, OR = odds ratio.

**FIGURE 3:**
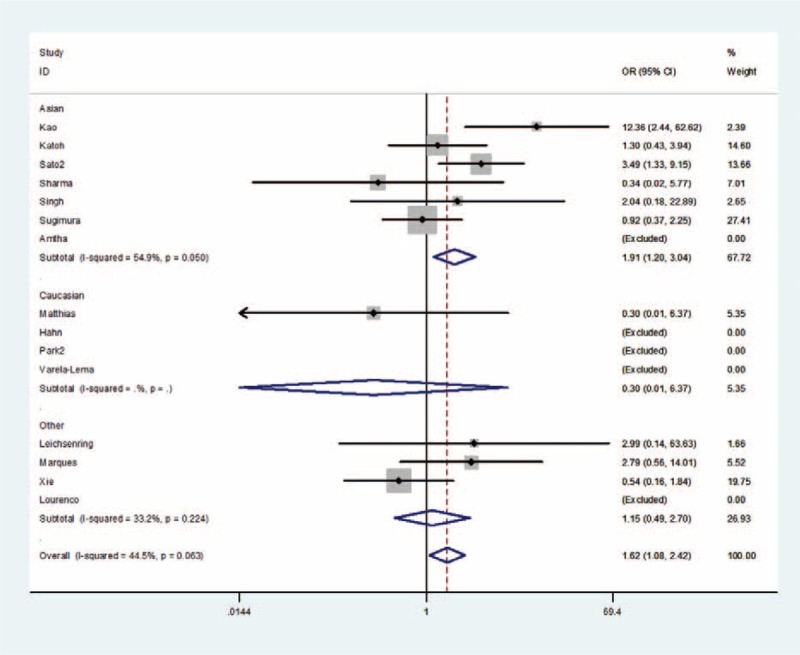
Forest plot of oral cancer risk related to CYP1A1 rs1048943 polymorphism in Asians under GG versus AA genetic model. CI = confidence interval, OR = odds ratio.

The results were shown in Tables 3 and 4. Overall, *CYP1A1* rs4646903 polymorphism was closely associated with the increased risk of oral cancer according to the pooled ORs (CC vs TT: OR 1.65, 95% CI 1.33–2.05; CC vs TC+TT: OR 1.77, 95% CI 1.48–2.11; C vs T: OR 1.17, 95% CI 1.07–1.28). Using the CC+TC versus TT model and the TC versus TT model, we did not find any significant association (Table 3). Subgroup analysis by ethnicity showed similar association of rs4646903 with oral cancer in Asians in the same genetic models tested (CC vs TT: OR 1.70, 95% CI 1.35–2.13; CC vs TC+TT: OR 1.83, 95% CI 1.52–2.20; C vs T: OR 1.17, 95% CI 1.06–1.29) but not in whites. Further subgroup analysis by source of control revealed that rs4646903 was significantly related with oral cancer susceptibility in hospital-based population (CC vs TT: OR 1.53, 95% CI 1.15–2.05; CC vs TC+TT: OR 1.67, 95% CI 1.26–2.20) and population-based population (CC vs TT: OR 1.81, 95% CI 1.31–2.51; CC+TC vs TT: OR 1.23, 95% CI 1.04–1.46; CC vs TC+TT: OR 1.84, 95% CI 1.46–2.32; C vs T: OR 1.26, 95% CI 1.09–1.46), as shown in Figure 2. For *CYP1A1* rs1048943, subgroup analysis by ethnicity indicated that it was related with increased risk of oral cancer in Asians (GG vs AA: OR 1.91, 95% CI 1.20–3.04; GG vs GA+AA: OR 1.76, 95% CI 1.10–2.80; G vs A: OR 1.27, 95% CI 1.07–1.50) but not in whites and other ethnic groups (Figure 3). However, no significant relationship was found between the *CYP1A1* rs1048943 polymorphism and oral cancer risk in overall analysis and subgroup analysis by source of control.

**TABLE 3:**
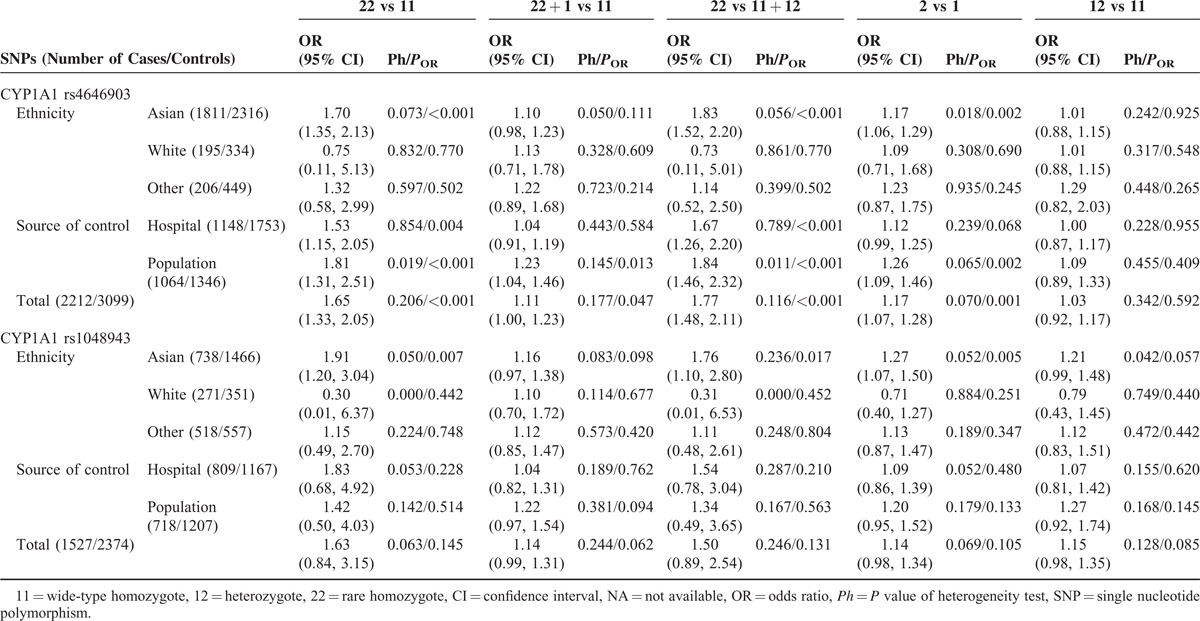
*CYP1A1* Polymorphisms and Oral Cancer Risk

**TABLE 4:**
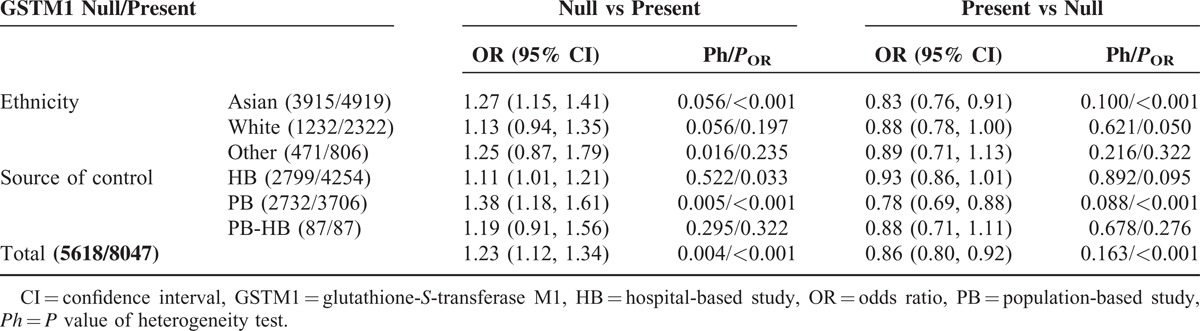
*GSTM1* Null/Present and Oral Cancer Risk

**FIGURE 4:**
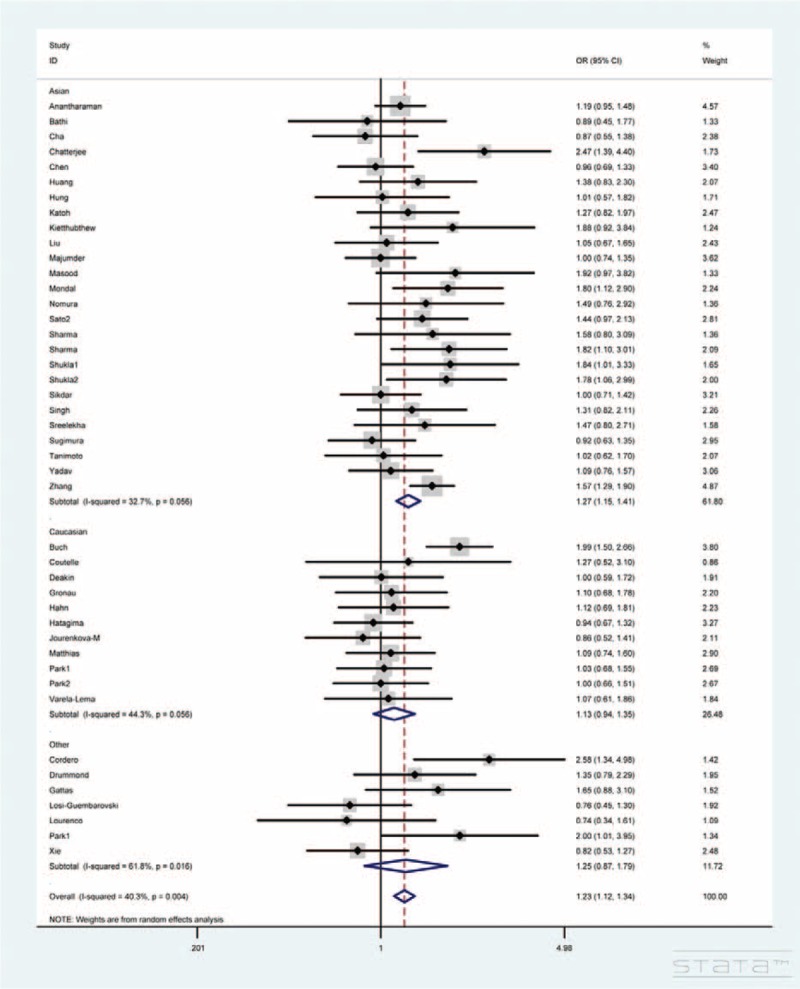
Forest plot of oral cancer risk associated with GSTM1 null/present. For each study, the estimates of OR and its 95% CI are plotted with square and a horizontal line. The area of the squares reflects the weight (inverse of the variance). The diamond represents the summary OR and 95% CI. GSTM1 = glutathione-*S*-transferase M1, CI = confidence interval, OR = odds ratio.

With respect to *GSTM1* polymorphisms, null genotype showed obvious relevance to oral cancer susceptibility (OR 1.23, 95% CI 1.12–1.34), especially in Asians (OR 1.27, 95% CI 1.15–1.41), compared with present genotype. Moreover, it was demonstrated that null genotype could affect individual susceptibility to oral cancer in both hospital- and population-based populations (OR 1.11, 95% CI 1.01–1.21; OR 1.38, 95% CI 1.18–1.61), as displayed in Figure 4.

### Sensitivity Analysis

Sensitivity analysis was performed to evaluate the influence of each individual study on the pooled ORs. The recalculated ORs were not substantially influenced, which suggested our results were stable.

### Publication Bias

**FIGURE 5:**
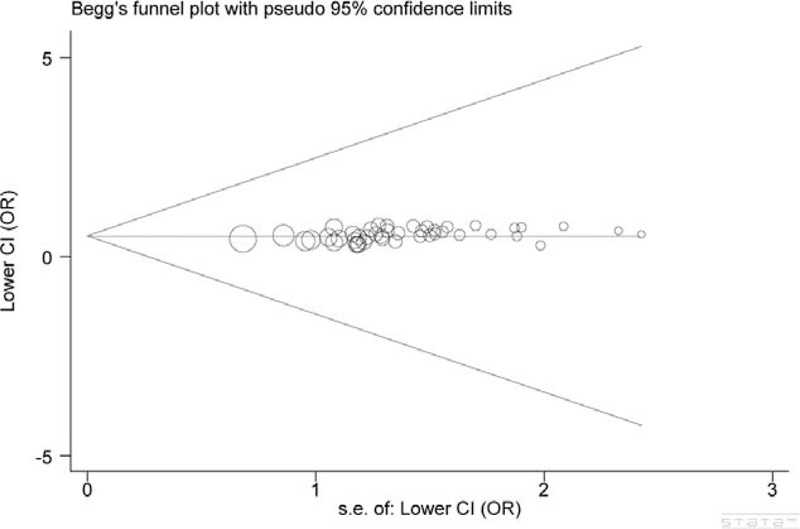
Begg funnel plot of publication bias. Each point represents a separate study for the indicated association. Log (OR), natural logarithm of OR; horizontal line, mean effect size. CI = confidence interval, OR = odds ratio.

Egger test and Begg funnel plot were conducted to estimate publication bias. The shape of the funnel plot was relatively symmetrical (Figure 5). Additionally, the result of Egger test did not show statistical evidence for bias (*P* = 0.656). Thus, there was no obvious publication bias in our meta-analysis, and the results were credible.

## DISCUSSION

Oral cancer has become a major health problem characterized by high incidence, poor survival rate, and severe functional and cosmetic defects accompanying the treatment.^70^ Moreover, it has been demonstrated that genetic and environmental factors could affect individual susceptibility to oral cancer. Therefore, it is significant to investigate the association of *CYP1A1* and *GSTM1* polymorphisms with oral cancer risk.

*CYP1A1* rs4646903 and rs1048943 polymorphisms contribute to increased enzyme activity of CYP1A1 and are crucial to the activation of PAHs.^6,39^ The null genotype of *GSTM1* polymorphism could result in the inactivation of GSTM1 enzyme and thus decrease the capacity of detoxifying carcinogens.^71^ So far, several epidemiological studies have evaluated the association of *CYP1A1* and *GSTM1* polymorphisms with oral cancer susceptibility. In our study, *CYP1A1* rs4646903 was verified to increase the risk of oral cancer, particularly in Asians, whereas CYP1A1 rs1048943 polymorphism did not show significant relationship with oral cancer susceptibility, when we pooled all data together, but demonstrated a statistically significant association when data were limited to Asians, which was consistent with the results of most previous studies.^2,24,37,40,45,47,53,56,58,71,72^ However, there were some studies with opposite results to ours. Among them, Losi-Guembarovski et al^51^ and Amtha et al^58^ found that there was no significant association between *CYP1A1* rs4646903 polymorphism and oral cancer risk. In the studies of Katoh et al^38^ and Sreelekha et al,^39^*CYP1A1* rs1048943 showed no association with the susceptibility of oral cancer. Compared with the above studies, our study showed advantages in population composed of Asians, whites, and other ethnic groups and relatively lager sample size, which make our result much more credible.

For the association between null genotype of *GSTM1* polymorphisms and oral cancer risk, the results were also not conclusive.^25–31,33,34,41,44,63,65,66,68,73,74^ Our meta-analysis demonstrated that null genotype of *GSTM1* polymorphisms was significantly associated with overall risk of oral cancer. However, the significance was lost in further analysis among whites.

The 3 polymorphisms analyzed in the present work have 1 thing in common. None of them demonstrated a significant association with genetic risk of oral cancer in whites. The null results may be biased because the current sample is insufficient to determine whether there is an association in this population. Another possibility is that both *CYP1A1* and *GSTM1* polymorphisms modify oral cancer risk in an ethnic-specific fashion due to different genetic backgrounds. These possibilities clearly require to be investigated in future research.

Certain limitations in our study should be noted. First, our study was not stratified by smoking status, which was identified as a key factor in oral cancer risk.^54^ Second, subgroup analysis of *CYP1A1* polymorphisms involved relatively fewer data in whites and other ethnic groups, which may produce some bias in the results. Finally, lack of original data about present genotype of *GSTM1* polymorphisms might influence the combined results.

In conclusion, our meta-analysis indicates that *CYP1A1* rs4646903, rs1048943, and null genotype of *GSTM1* polymorphisms are possible risk factors for oral cancer, especially in Asians. In the future, in-depth studies are required to further explore the association.
